# Cryo-EM Structure and Activator Screening of Human Tryptophan Hydroxylase 2

**DOI:** 10.3389/fphar.2022.907437

**Published:** 2022-08-15

**Authors:** Kongfu Zhu, Chao Liu, Yuanzhu Gao, Jianping Lu, Daping Wang, Huawei Zhang

**Affiliations:** ^1^ Department of Biomedical Engineering, Southern University of Science and Technology, Shenzhen, China; ^2^ Cryo-EM Facility Center, Southern University of Science and Technology, Shenzhen, China; ^3^ Department of Child and Adolescent Psychiatry, Shenzhen Kangning Hospital, Shenzhen Mental Health Center, Shenzhen, China; ^4^ Department of Orthopedics, Shenzhen Intelligent Orthopaedics and Biomedical Innovation Platform, Guangdong Provincial Research Center for Artificial Intelligence and Digital Orthopedic Technology, Shenzhen Second People’s Hospital, The First Affiliated Hospital of Shenzhen University, Shenzhen, China; ^5^ Guangdong Provincial Key Laboratory of Advanced Biomaterials, Southern University of Science and Technology, Shenzhen, China

**Keywords:** serotonin, psychological disorders, TPH2, virtual screening, MD simulation

## Abstract

Human tryptophan hydroxylase 2 (TPH2) is the rate-limiting enzyme in the synthesis of serotonin. Its dysfunction has been implicated in various psychiatric disorders such as depression, autism, and bipolar disorder. TPH2 is typically decreased in stability and catalytic activity in patients; thus, screening of molecules capable of binding and stabilizing the structure of TPH2 in activated conformation is desired for drug development in mental disorder treatment. Here, we solved the 3.0 Å cryo-EM structure of the TPH2 tetramer. Then, based on the structure, we conducted allosteric site prediction and small-molecule activator screening to the obtained cavity. ZINC000068568685 was successfully selected as the best candidate with highest binding affinity. To better understand the driving forces and binding stability of the complex, we performed molecular dynamics simulation, which indicates that ZINC000068568685 has great potential to stabilize the folding of the TPH2 tetramer to facilitate its activity. The research might shed light on the development of novel drugs targeting TPH2 for the treatment of psychological disorders.

## Introduction

The biogenic monoamine serotonin (5-hydroxytrptamine, 5-HT), defined as a neurotransmitter or a hormone, has been implicated in various physiological functions ranging from cell growth and development to metabolic processes (Weaver et al., 2016; Okaty et al., 2019; Bacqué-Cazenave et al., 2020). Many psychiatric disorders such as depression, autism, and bipolar disorder, as well as Alzheimer’s disease, are closely associated with the dysregulation of the serotonin secretion (Mansour et al., 2005; DeFilippis and Wagner, 2014; Kraus et al., 2017; Chakraborty et al., 2019; Takumi et al., 2020). Serotonin is also reported to play important roles in inflammatory, osteoporosis, gastrointestinal, and cardiovascular diseases (Ayme-Dietrich et al., 2017; D’Amelio and Sassi, 2018; Balakrishna et al., 2021). The regulation of the serotonin signaling, recycling, and degradation has emerged to be potential targets for the therapy of related diseases (Bader, 2020).

Serotonin is synthesized from tryptophan by tryptophan hydroxylase (TPH) and subsequent aromatic amino acid decarboxylase (AADC) (González-Castro et al., 2014; Zhang, 2016). AADC also catalyzes the formation of other monoamines and thus is not specific for serotonin synthesis (Montioli et al., 2020; Montioli and Borri Voltattorni, 2021). By contrast, TPH is specific and acts as the rate-limiting enzyme for serotonin synthesis and is considered as a crucial target for the regulation of the serotonergic system (Swami and Weber, 2018). There are two distinct TPH homologies in humans, TPH1 and TPH2, which are separately located on chromosomes 11 and 12, respectively, sharing 71% sequence identity in amino acids (McKinney et al., 2005). TPH1 and TPH2, together with phenylalanine hydroxylase (PAH) and tyrosine hydroxylase (TH), form the family of pterin-dependent aromatic amino acid hydroxylases (AAAHs) that catalyze the hydroxylation of their respective aromatic amino acid substrates in a conserved mechanism, with molecular oxygen, tetrahydrobiopterin (BH4), and Fe^2+^ as cofactors (Patel et al., 2016; Waløen et al., 2017). The members of AAAHs are all similarly composed of three domains in structures: an N-terminal regulatory domain for robustly modulating its activity, a catalytic domain for substrate binding, and a C-terminal domain for maintaining the oligomerization states (Cao et al., 2010).

The oligomerization state is critical for the activities of AAAH families. The C-terminal domain is important for their oligomerization. Previous studies have shown that the deletion of C-terminal residues will decrease and nearly abolish the activities of TPH2 (Tenner et al., 2007). The addition of phenylalanine will shift the state of the TPH2 variant from monomer to dimer and change its activity by threefold (Tidemand et al., 2016). In pathological conditions such as Parkinson’s disease, TPH2 may form disulfide-bonded aggregates upon oxidation and eventually affect its activity (Kuhn et al., 2011). Studies on PAH have also shown that PAH exists in the solution as a dimer and two architecturally distinct tetramers, while its substrate phenylalanine is involved in the regulation of PAH states and affect its activity (Arturo et al., 2016; Flydal et al., 2019). Patel et al. (2016) also reported that phenylalanine can bind to the dimerization interface and regulatory domain of PAH and regulate its activity (Patel et al., 2016). A previous report on human TH showed that it exists as enzymatically stable tetramers and octamers in the solution, and missense mutations on the interface will disrupt its oligomeric states, decrease its activity, and eventually cause disease such as DOPA-responsive dystonia (Szigetvari et al., 2019).

Although TPH1 and TPH2 are highly conserved in both structural and catalytic mechanisms, there are many differences in their phosphorylation sites, expression patterns, and physiological processes (McKinney et al., 2005). TPH1 is dominantly expressed in the enterochromaffin cells of the gut epithelium, where serotonin is synthesized and taken up by platelets via serotonin transporters (Schoenichen et al., 2019). TPH1 also functions in other tissues such as the lung, pancreas, and kidney as well as the pineal gland, where serotonin is synthesized as a precursor for melatonin, a hormone that functions in sleep and pain (Coon et al., 1996; Côté et al., 2003; Walther and Bader, 2003). Most of the circulating serotonin is deviated from TPH1 but not TPH2 (Gershon, 2013). TPH2 is dominantly expressed in the central nervous system, where TPH1 is not expressed (Walther et al., 2003; Patel et al., 2004). TPH2 was found in the Raphe nuclei of the brain stem and plays multiple roles in neurometabolic and neuropsychiatric disorders (Liu et al., 2021). A small amount of TPH2 is expressed in the enteric nervous system, where it functions similarly as TPH1 (Neal et al., 2009; Li et al., 2011).

The serotonin level is decreased in the brain; meanwhile, its level is increased in peripheral blood in most psychiatric disorders (Gabriele et al., 2014; David and Gardier, 2016), indicating that the activity of THP2 is decreased and the activity of TPH1 is increased. Thus, an activation on TPH2 and inhibition on TPH1 are desired. Inhibitors targeting TPH1 have been designed and developed for a long time (Engelman et al., 1967; Stokes et al., 2000; Zimmer et al., 2002). However, owing to the extremely low stability of TPH2, the structural and biochemical characterization of TPH2 has not been revealed for a long time (D'Sa et al., 1996; McKinney et al., 2004). In our report, we determined the cryo-EM structure of human TPH2 in tetrameric conformation at 3.0 Å resolution. After that, we carried out allosteric site prediction and small-molecule screening using the virtual screening technology. ZINC000068568685 (Cmpd 1) was successfully selected as the best candidate with highest score of −10.8 kcal/mol. To get more insight into the driving forces and binding stability of the complex, we performed molecular dynamics (MD) simulation, which indicates that Cmpd 1 has great potential to stabilize the formation of the TPH2 tetramer to facilitate its activity. Our research might shed light on the development of novel drugs targeting TPH2 for the treatment of mental disorders.

## Materials and Methods

### Gene Cloning, Protein Expression, and Purification

The full-length human TPH2 gene was purchased from Sino Biological Co., Ltd, and reconstructed to pCAG with an N-terminal Twin-Strep-tag and a 3×Flag-tag. The construct was then transfected into Expi293F (Thermo Scientific) with PEI reagent and cultured for 72 h at 37°C under 8% CO_2_. After that, cells were harvested, resuspended, and lysed by sonication in buffer A (50 mM HEPES pH 7.5, 150 mM NaCl, 0.1 mM FeSO4, 0.1 mM tryptophan, 0.1 mM EDTA, 10% v/v glycerol, 2% Tween-20, and 1 mM PMSF). Insoluble material was removed by centrifugation at 15,000 g and the supernatant was loaded on a 2 ml Strep-Tactin®^XT^ column equilibrated with lysis buffer. The column was washed successively with 2 mM ATP in buffer A to remove the endogenously expressed HSP70 protein before TPH2 was eluted in steps with three times of buffer A containing 5, 25, and 50 mM biotin. Fractions containing pure TPH2 protein were identified using SDS-PAGE and further purified using size exclusion chromatography. TPH2 was loaded on a Superose™ 6 Increase 10/300 GL column attached to an AKTA pure system (Cytiva) equilibrated in buffer B (50 mM HEPES pH 7.5, 150 mM NaCl, 0.02% w/v glyco-diosgenin). Fractions were assessed using SDS-PAGE and concentrated for cryo-EM analysis. Approximately 0.25 mg of full-length TPH2 can be obtained from 500 ml of cells.

### Cryo-Electron Microscopy

The freshly purified TPH2 was used to prepare cryo-EM grids. A drop of 4 μl TPH2 solution at the concentration of about 2.5 mg/ml was loaded to the holey film grid (Ni-Ti R2/2, 300 mesh). The grid was glow-discharged prior to sample loading and then blotted for 2.5 s under 100% humidity at 4°C using Vitrobot Mark IV (Thermo Scientific). After that, the grid was plunged into liquid ethane, which was precooled by liquid nitrogen. The grid was then observed using a Titan Krios microscope (Thermo Scientific) operated at 300 kV and equipped with a K2 Summit camera (Gatan). All images were recorded automatically using SerialEM under a nominal defocus value ranging from −1.5 to −2.5 μm and a nominal magnification of ×165 k, corresponding to a pixel size of 0.842 Å. Each micrograph was dose-fractionated to 32 frames with 0.1125 s exposure time in each frame. The dose rate was 1.1 counts per physical pixel per second, corresponding to 1.5625 electrons per square angstrom per second.

### Cryo-EM Image Processing

For all micrographs, motion correction was carried out immediately after data collection using the MotionCor2 program (Zheng et al., 2017). After that, 5,559 micrographs were imported to RELION 3.1.2 (Scheres, 2016) for further processing. CTFFIND 4.1 was applied to evaluate the defocus parameters (Rohou and Grigorieff, 2015). A total of 1,416,664 particles were picked using Gautomatch with the template of the crystal structure of PAH (PDB entry: 5DEN). Particles were imported into cryoSPARC for further analysis. After several rounds of 2D classification, 486,660 particles remained for 3D classification. One of the best 3D classes with the highest quality in resolution was selected and performed for refinement and postprocessing, which resulted in a final map at 3.0 Å overall resolution with D2 symmetry. The resolution was estimated using the gold-standard Fourier shell correlation at the cutoff value of 0.143. All 3D reconstruction structures were visualized using Chimera 1.15 (Pettersen et al., 2004).

### Model Building, Refinement, and Validation

The cryo-EM model of TPH2 was primarily generated in PHNIX (Adams et al., 2010) by docking the crystal structure of human TPH2 (PDB ID 4V06) into our map and then manually revised in Coot (Emsley and Cowtan, 2004). After that, the model was refined using the real-space refinement module in the Phenix program and subsequently fixed manually in Coot. At last, the model was validated using the MolProbity tool in Phenix. The figures of the model were visualized and prepared in PyMOL (Barber, 2021) and Chimera. Structural analysis was performed using LigPlot+ (Laskowski and Swindells, 2011).

### Allosteric Site Prediction

The allosteric site prediction was performed using the CavityPlus web server (Xu et al., 2018). First, we input the structure of the TPH2 tetramer and applied the cavity program to detect the potential cavity and ranked them using druggability scores. Based on the detected cavities, the submodule CorrSite 2.0 program was used to identify potential allosteric ligand binding sites. Allosteric site prediction was performed using default parameters.

### AutoDock Dataset Acquisition

The ZINC15 database (Istifli, 2020) provides a large quantity of small molecules. We downloaded approximately 180,000 drug compounds as the data list based on their log P and pH values. All these compounds are standard, in-stock, neutral lead-like small molecules, based on which we can use relevant software to decide their protonation states and add hydrogen atoms and charges so as to do subsequent procedures for molecular docking.

### Molecular Docking

After the three-dimensional structure of TPH2 was determined using cryo-EM, its protonation state was obtained, H atoms were added, and atomic radius was assigned using AutoDock prior to molecular docking. Then, following the AutoDock algorithm, we added nonpolar hydrogen atoms to heavy atoms and docked small molecules into the target protein using the AutoDock Vina v1.2.0 program (Koebel et al., 2016; Vieira and Sousa, 2019). The Gasteiger partial charge (Bikadi and Hazai, 2009; Mittal et al., 2009) was used, and the active pocket was chosen to just cover the vital amino acid identified in the allosteric site prediction step (Jain, 2006). The grid spacing was hereby set to 0.508 Å with a box size of 50 × 48 × 47 to be the active pocket. In total, 10 docking modes were set for each molecule, and the best one was kept for MM/PBSA calculations. The chosen mode had the expected lowest binding affinity. We used an MPI-based parallel implementation of the AutoDock Vina program VinaLC (Zhang et al., 2013) for a large quantity of docking computations.

### Energy Minimization Step

Energy minimization, which requires the location of the simulation system’s energy minimum, is a key determining step before MD. It tries to decide the most stable molecular 3D structure under the specified potential to ensure that steric hindrance or the geometric structure is excluded. Afterward, the solvent and charge are added. Then, the steepest descent method is chosen as the algorithm (Donnelly et al., 2021).

### Equilibration Procedure

Before MD simulation begins, we should do two equilibration procedures: NVT equilibration and NPT equilibration. Under an NVT ensemble, temperature is supposed to reach maximum toward the desired value. Velocity-rescale is chosen as the heat-bath algorithm. NPT equilibration is implemented under an NPT ensemble, and Parrinello–Rahman is selected as the pressure-bath algorithm. Periodic boundary conditions are applied to both equilibrations. The simulation durations are both 100 ps.

### Molecular Dynamics Simulation

The 3D coordinates were obtained from the TPH2 cryo-EM structure at 3.0 Å resolution. Then, the MD simulation was implemented using the Gromacs 2019.6 software package (Faccioli et al., 2012; van der Spoel et al., 2012; Zhang et al., 2019) with the AMBER99SB-ILDN force field (Somavarapu and Kepp, 2015; Ouyang et al., 2018) for the protein and the TIP3P force field (Ong and Liow, 2019) for the water solvent. The appropriate number of sodium counter ions were added to neutralize the system. VMD (Humphrey et al., 1996; Fernandes et al., 2019) and XMGRACE software programs (Machné et al., 2006; Saranya Ganesh et al., 2020; Yalameha et al., 2022) were employed to visualize the molecules.

Here, we introduce the root-mean-square deviation (RMSD) indicator, which quantitatively assesses the difference between the target structure and the reference structure. This value can recognize large protein structure changes from the beginning point. A levering off of this curve usually reveals protein stabilization.

$$RMSD=\sqrt{\frac{\sum_{j=0}^{N}\left[m_{j}*{\left(X_{j}-Y_{j}\right)}^{2}\right]}{M}}$$

Another numerical value similar to RMSD that usually measures the spatial changes of biomolecules is root-mean-square fluctuation (RMSF). RMSF is a per-residue or per-atom quantity that describes each residue’s or atom’s change over the whole trajectory. It measures each individual residue or atom flexibility, or how much a specific residue or atom vibrates over the simulation course.

$$RMSF=\sqrt{\frac{1}{T}\sum_{t=1}^{T}\sum_{j=1}^{N}{\left(x_{j}\left(t\right)-\bar{x_{j}}\right)}^{2}}$$

The radius of gyration (Rg) is commonly described as the imaginary distance from the centroid. It describes the compactness of a protein, which can be formulated as follows:

$$k=\sqrt{\frac{I}{m}}$$

Prior to computing the above three parameters, we treat the MD trajectories and make sure no periodic boundary condition is applied.

### MM/PBSA Energy Decomposition

The free energy of binding (ΔG_*bind*_) is used to judge how a ligand changes from the solvated mode to the protein-bound mode, and it is supposed to be a large negative free energy. In thermodynamics, this term consists of the enthalpic change (ΔH) and the entropic change (ΔS).

$$\Delta G_{bind}=\Delta H-T\Delta S$$

The values for ΔH on the right-hand side can be decomposed into three terms:

$$\Delta H=\Delta E_{MM}+\;\Delta G_{PB}+\;\Delta G_{np}$$

Therefore, ΔG_*bind*_ can be rewritten as

$$\Delta G_{bind}=\;\left(\Delta E_{MM}+\;\Delta G_{PB}+\;\Delta G_{np}\right)-T\Delta S$$

In this equation, ΔG_np_, ΔG_PB_, and ΔE_MM_ denote the nonpolar solvation energy, the Poisson–Boltzmann energy, and the molecular mechanics energy, respectively. ΔG_np_ + ΔG_PB_ are combined as ΔG_PBSA_. ΔE_MM_ consists of internal, van der Waals, and Coulombic energies.

For the MM/PBSA computation, the chief aim is to identify important residues that bind most closely to the corresponding protein. The nonpolar solvation free energy follows a linear relationship with the solvent-accessible surface area (SASA):

$$\Delta G_{np}=\gamma SASA+\beta$$

In this calculation, we set the force field for the protein as amber99sb-ildn and the ligand force field as generalized AMBER force field (GAFF) (Ozpinar et al., 2010) to make energy decomposition.

## Results and Discussion

### Purification and Structure Determination of Tryptophan Hydroxylase 2 Tetramer

Determining the oligomeric states and atomic models of TPH2 is critical to understand its physiological roles and to develop novel interventions. The crystal structure of the TPH2 catalytic domain has been deposited in the Protein Data Bank previously (PDB: 4V06). However, there is no associated publication available, and thus, detailed description is lacking. In the crystal structure, TPH2 is deposited as a dimer form in the asymmetric unit. However, when symmetry and crystal packing were considered, a possible tetramer form can be found in the unit cell, but it is still hard to tell its oligomeric states in the solution. Thus, we set out to determine the solution structures of human TPH2 using the cryo-EM method using its full-length form.

**FIGURE 1:**
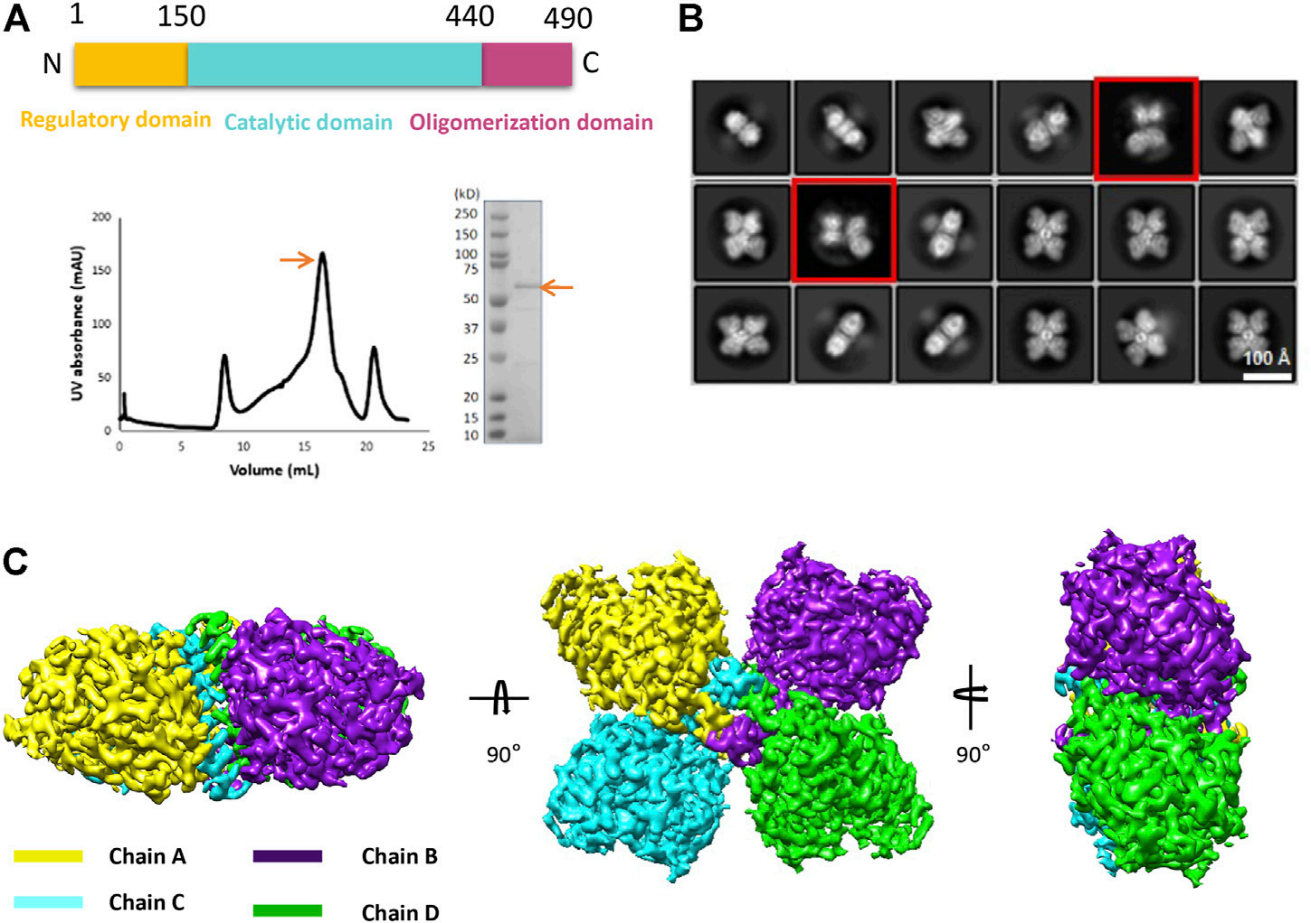
Purification and structure determination of human tryptophan hydroxylase 2 (TPH2). **(A)** domain organization, size exclusion chromatography profile, and SDS-PAGE analysis of TPH2 in peak fraction. The target items are indicated with arrows. **(B)** 2D class average of cryo-EM micrographs of TPH2. The red box indicates the TPH2 asymmetric tetramer in a dynamic state. **(C)** cryo-EM 3D map of TPH2 tetramer in different views and colored by chain.

Using the optimized expression and purification methods, we successfully obtained the full-length human TPH2 in given conditions. The size exclusion chromatography profile and the SDS-PAGE result displayed in Figure 1A indicate that the obtained protein is at high purity and in the monodisperse oligomerization state. After several rounds of cryo-EM sample preparation, image collection, and data processing, we finally determined the structure of TPH2 in its tetrameric conformation at 3.0 Å resolution (Supplementary Figure S1, Table 1, EMDB-32540, PDB: 7WIY). The representative 2D classes in Figure 1B show that the symmetric tetramer is the predominant form, while there are also a small population of tetrameric particles without symmetry, and the four monomers are not precisely the same (with red box), which might indicate several potential transition states between different assemble states. However, we failed to determine the three-dimensional structures in high resolution for those special states because of its small population and insufficient projections in 2D classes. To boost the quality of the map, only the particles with clear symmetry were selected during the data processing. The final postprocessed 3D map is shown in Figure 1C. TPH2 is assembled by four protomers in a D2 symmetry, which is different from the previously deposited crystal structure in dimer form.

**TABLE 1:** Summary of data collection, processing, and atom model statistics.

Data collection	
EM equipment	Titan Krios (Thermo Fisher)
Voltage (kV)	300
Detector	Gatan K2 Summit
Energy filter	Gatan GIF, 20 eV slit
Pixel size (Å)	0.842
Total Electron dose (e-/Å2)	50
Defocus range (µm)	−1.5 to −2.5
3D Reconstruction	
Software	Relion/CryoSPARC
Number of micrographs	5559
Final particles	110,963
Symmetry	D2
Final resolution (Å)	3.0
Map sharpening B-factor (Å^2^)	172.5
Refinement	
Software	Phenix
Model composition	
Protein residues	240 × 4
ligand	4 Fe^2+^; 4 IMD
R.M.S. deviations	
Bonds length (Å)	0.009
Bonds Angle (˚)	0.707
Ramachandran plot statistics (%)	
Preferred	90.61
Allowed	9.39
Outliers	0

### Overall Structure Analysis of Tryptophan Hydroxylase 2

**FIGURE 2:**
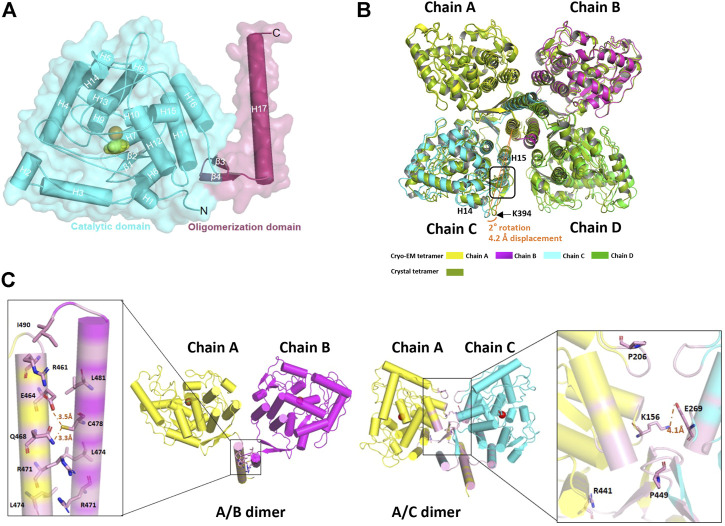
Structure analysis of human tryptophan hydroxylase 2 (TPH2). **(A)** cartoon display of TPH2 monomer. The catalytic domain is colored cyan, while the oligomerization domain is colored warm pink. **(B)** superposition of the cryo-EM structure with the crystal structure of TPH2 (PDB entry: 4V06). The cryo-EM structure of TPH2 in tetramer is colored by chain, while the crystal structure is colored split pea. The tetramer of the crystal structure is obtained by dimer using the symmetry option. The arrow and curve indicate the different secondary structures and the rotation they adapted. **(C)** interfaces analysis of TPH2 dimers. The left shows the interface of chain A and chain B, and the right shows the interface of chain A and chain C. The interface is shown in light magenta. Key residues across interfaces of chain A/B and chain A/C are shown as sticks.

The structure of TPH2 is dominantly formed with helices. As shown in Figure 2A, there are 17 helices (labeled as H1–H17) and 4 β sheets in the TPH2 monomer, together with Fe^2+^ and imidazole as cofactors. The TPH2 monomer contains the catalytic domain and oligomeric domain but lacks the N-terminal regulatory domain, although full-length TPH2 was used for sample preparation. Since the affinity tag used for purification was located in the N-terminus and the protein can be successfully purified, we speculate that TPH2 exists as a full-length version in the sample solution and the N-terminus is not degraded. Besides, the outcomes of SDS-PAGE also indicate that the molecular mass of the sample is in agreement with the full-length ones. Thus, it is most likely that its N-terminal regulatory domain is too flexible to align the signal during 3D construction and thus is not seen in the final map. The crystal structure (PDB:4V06) also misses the N-terminal domain, resembling the cryo-EM structure with the RMSD value at 0.789 Å when chain A of each model was aligned, although the crystal structure is in dimer form in the asymmetric unit as deposited and the cryo-EM structure is in tetramer form. Using the symmetry option, we prepared the tetramer form of TPH2 based on the crystal structure and superimposed it with the cryo-EM tetramer structure with chain A as the reference. We found that the chain C–D of the crystal structure and cryo-EM structure shows about 2° rotation (with reference to the central oligomerization helix domain) and about 4.2 Å displacement (with reference to Cα from K394) (Figure 2B). This observation is similar to that in a previous report that different conformational oligomers of human phenylalanine hydroxylase show a rotation of ∼3° and displacements up to 3 Å (Flydal et al., 2019). Besides, the secondary structure of the loops between H14 and H15 was transformed to three pairs of β sheets in the crystal structure (inside the rectangle box in Figure 2B). The cryo-EM model seems to fold less tightly than the crystal structure and shows more flexible and dynamic properties. A previous report showed that the binding of the N-terminal regulatory domain to the catalytic domain will inhibit TPH2 activity and thus acts as a negative regulator (Tenner et al., 2007). The deletion of the N-terminal domain has also been demonstrated to abolish its inhibitory effect (Tenner et al., 2007). Therefore, both the crystal structure and the cryo-EM structure might represent the activated state of TPH2, because their N-terminal is not bound closely to the catalytic domain whether it resulted from truncation or flexibility.

### Interface Analysis of the Tryptophan Hydroxylase 2

The oligomeric state of the AAAH protein family has been reported to be essential for their function (Flydal and Martinez, 2013). For example, the deletion of the C-terminal 19 amino acids of TH leads to 70% reduction of enzyme activity (Walker et al., 1994). Likewise, the removal of the last 51 amino acids of TPH2 also dramatically interferes with the tetrameric conformation and nearly abolishes the activity of TPH2 (Tenner et al., 2007). To better understand the assembly mechanisms of TPH2 oligomerization, we performed the interface analysis of the TPH2 tetramer. Two types of interfaces were identified and shown in Figure 2C. For the A/B interface, the C-terminal helix 17 of each chain forms a leucine zipper–like motif to maintain their interaction. The interface area is calculated to be 882 Å^2^, involving 13 amino acids of each chain such as R461, R471, L474, L481, and I490. However, it gets complicated for the A/C interface, which forms a much tighter and consolidated interface. The interface area expands to be approximately 1,838 Å^2^, and there are 27 amino acids participating in the interaction, located at not only helix 17 but also helix 11/15/16, as well as the antiparalleled β3/β4 sheets and some nearby loops. Nonbonded contact including the hydrophobic effect and van der Waals forces plays the most essential roles. The mutants of those represented residues such as R441H and P449R are the most deleterious factors impairing the stability of TPH2 and are prevalent in depression, bipolar disorder, and autism patients. P206 is also directly involved in the hydrophobic environment and affects the stability of the A/C interface, which explains the catastrophic effect of the P206S mutant in a previous report (Cichon et al., 2008). Besides, we also identified three hydrogen bonds mediated by E464, E468, and C478 in the A/B interface and a stable salt bridge between K157 and E269 in the A/C interface, which certainly contribute to the oligomeric assembly. Therefore, understanding the assembly mechanism of TPH2 may be beneficial to reveal the catalytic processes, explaining and predicting the deleterious mutants in patients.

### Decoding of Tryptophan Hydroxylase 2 Catalytic Mechanism

**FIGURE 3:**
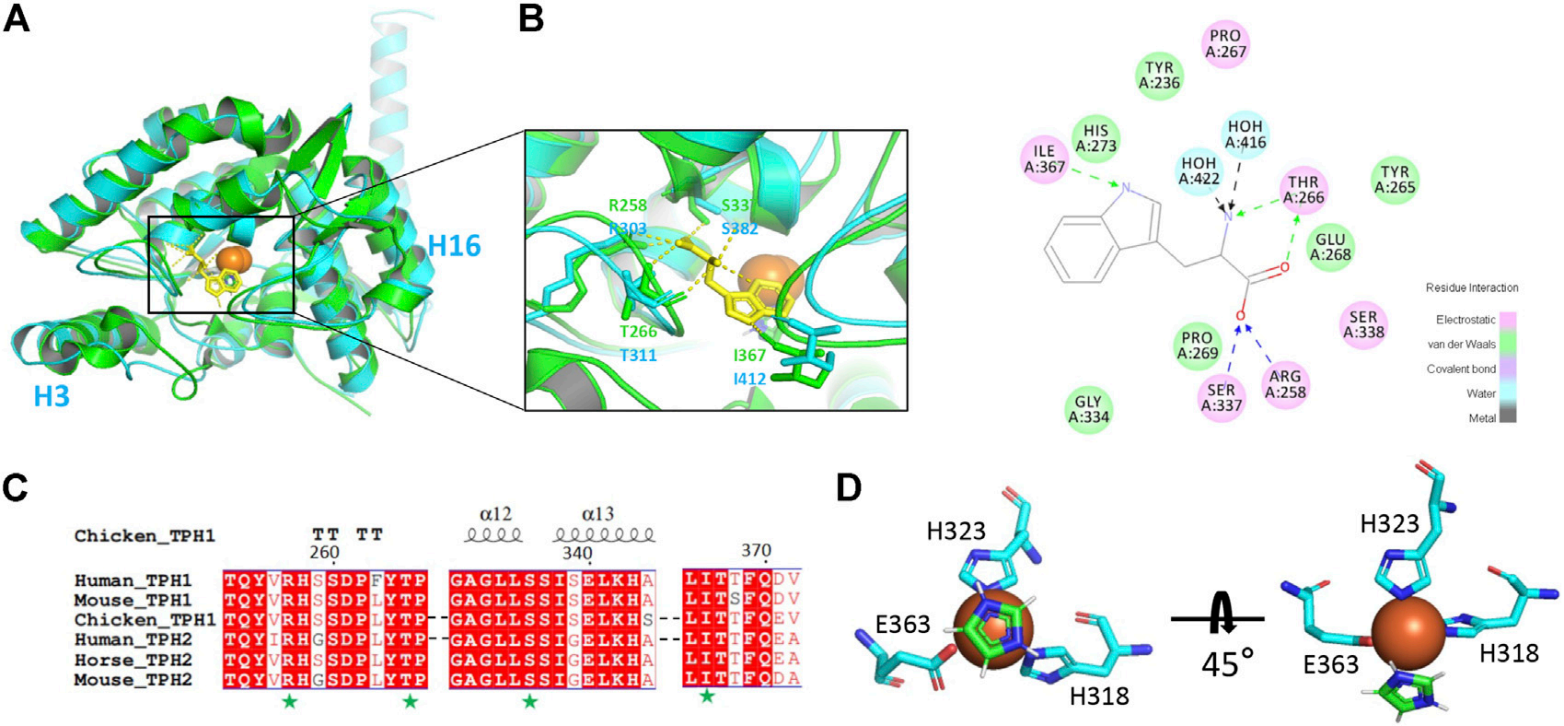
Cofactor and substrate binding site analysis. **(A)** superimposition of human tryptophan hydroxylase 2 (TPH2) with chicken TPH1 and substrate binding environment analysis. The human TPH2 is in cyan, and the chicken TPH1 is in chartreuse. The substrate tryptophan is depicted as sticks in yellow. The residues participating in binding are shown as sticks in corresponding colors. **(B)** schematic graph of all the residues interacting with tryptophan of chicken TPH1 is displayed. Electrostatic interaction is shown in pink, van der Waals interaction is shown in green, and water–ligand interaction is shown in cyan. **(C)** primary sequence alignment of human TPH2 and chicken TPH1 produced by MULTALIN and Esprit, in which amino acids with red background represent identity. The secondary structure of chicken TPH1 is depicted on the top, and the substrate binding sites are pointed below with a green pentagram. **(D)** display of Fe^2+^ and imidazole binding environment.

To get insight into the catalytic mechanism of TPH2, we superimposed and compared the obtained cryo-EM structure of human TPH2 with that of chicken TPH1, which is the only reported structure of TPH with substrate binding (PDB: 3E2T) (Windahl et al., 2008). As shown in Figure 3A, they highly resemble each other in whole although there are movements of H3 and H16 for TPH2. Figure 3B shows that R258, T266, S337, and I367 of chicken TPH1 determine the tryptophan binding specificity, and two water molecules around also contribute to the correct orientation of the substrate. The four residues also match well with the counterpart positions R303, T311, S382, and I412 in TPH2. Further analysis of the four residues also explains the disastrous effects of variants R303W and S383F of TPH2 in patients as in a previous report (Pereira et al., 2020). R303W could immediately reduce the substrate binding affinity and specificity. In contrast, S383F may impair the stability and mobility of the catalytic domain and thus destroy the substrate binding by changing the configuration of S382, according to a previous MD study (Pereira et al., 2020). To further investigate the potential substrate binding sites of human TPH2, we performed sequence alignment of the substrate binding regions using TPHs from different species including human, chicken, mouse, and horse, with reference to the binding site analysis of tryptophan in chicken TPH1. The results shown in Figure 3C indicate that the four functional residues are quite conserved across different species. It is possible that residues R303, T311, S382, and I412 are involved in the substrate binding in human TPH2. Furthermore, we performed interaction analysis of the Fe^2+^ cofactor with the active site of the TPH2 cryo-EM structure. Figure 3D shows that catalytic iron binds to conserved H318, H323, and E363, which form a 2-His-1-carboxylate facial triad, in a similar manner to that observed in other AAAHs enzymes. The nearby density can fit well with imidazole, and this is in agreement with the crystal structure, although the orientation of imidazole is slightly different. Further studies on decoding the catalytic mechanism are essential and may facilitate the design and development of molecules to stabilize and increase the activity of TPH2.

### Small-Molecule Screening

**FIGURE 4:**
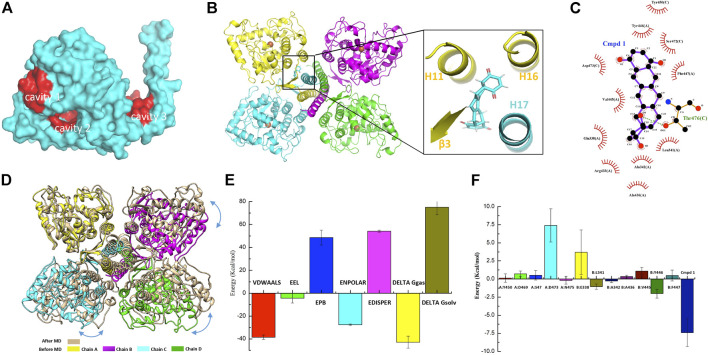
Small-molecule screening based on the cryo-EM structure of tryptophan hydroxylase 2 (TPH2). **(A)** representative cavities of TPH2 predicted using the CavityPlus web server. The cavity zones with high scores for allosteric sites are colored red. **(B)** cartoon display of TPH2 tetramer binding with the small-molecule Cmpd 1. Structure of Cmpd 1 was shown as sticks in the insert. **(C)** key residues are shown in the interaction analysis of Cmpd 1 and TPH2. **(D)** Structural alignment and conformational changes of Cmpd 1–bound TPH2 structures before and after simulation. Double arrow curves in blue color indicate the rotation of chains B–D with chain A as reference. **(E)** illustration of energy difference for the receptor–ligand complex. VDWAALS denotes the van der Waals energy, EEL denotes the electrostatic energy, ENPOLAR denotes the nonpolar solvation energy, and EPB denotes the Poisson–Boltzmann energy. **(F)** per-residue decomposition energies for the TPH2 with Cmpd 1 bound after simulation using MM/PBSA.

As discussed before, the oligomerization of TPH2 affects its activity dramatically, so we conducted computer-aided drug design based on the cryo-EM structure of the TPH2 tetramer. Prior to that, we carried out the allosteric site prediction of TPH2 using the CavityPlus web server, which lists the potential cavities according to the scores of druggability. Those top candidates are typically distributed to three zones as indicated in Figure 4A. Cavity 1 and cavity 2 zones are involved in substrate and cofactor binding, where binding of small molecules would inhibit its activity in theory. Cavity 3 shows the highest score of druggability, and the binding of a ligand might promote and stabilize the formation of the tetramer state, indicating it is more suitable for drug screening. Therefore, we used virtual screening technology based on cavity 3 with the ZINC15 database where we selected approximately 180,000 small molecules to screen the most desired candidate to bind and stabilize the tetramer conformation of TPH2. Supplementary Table S1 shows the top 20 ZINC IDs and their binding affinity to TPH2. The binding affinity defines the favor of a ligand to the receptor. The larger the negative value is, the stronger binding force it illustrates. As shown in Figure 4B, Cmpd 1 possesses the most negative binding affinity at −10.8 kcal/mol and is conceived as the best ligand. The predicted complex of the Cmpd 1 with the TPH2 tetramer after docking is shown in Figure 4B, where the protein is shown as a cartoon and the ligand is displayed in sticks. The capture suggests that the small-molecule Cmpd 1 fits well with the given cavity. Cmpd 1 was coordinated by helices H11, H16, and H17 and sheet β3. Further analysis using LigPlot + reveals that residues E338, L341, A342, A436, R433, V445, Y446, and F447 from chain A, together with residues Y450, S472, D473 and T476 from chain C, may be involved in the binding of Cmpd 1 to TPH2 (Figure 4C).

### Energy Minimization, Equilibration, and Unrestrained Molecular Dynamics Simulation

Energy minimization and equilibration are performed before conducting MD simulation. The topology file was produced by AMBER99SB-ILDN for the TPH2 tetramer and TIP3P for water. As illustrated in Supplementary Figure S2A, the initial potential energy is −5.17 × 10^5^ kJ/mol, which descends steeply and stabilizes at 5.16 × 10^6^ kJ/mol after 2,500 steps of minimization. Supplementary Figure S2B shows that the system was heated gradually in the NVT ensemble to 300 K in 100 ps followed by EM. The original velocities corresponding to the starting temperature were assigned from a Maxwellian distribution. Afterward, we performed the pressure equilibration, as shown in Supplementary Figure S2C. The input file is generally similar to the parameter file of NVT equilibration. The Parrinello–Rahman barostat is exerted to the pressure coupling section. In the process of NPT equilibration, the mean value of pressure was 0.8 ± 59.3 bar, and the reference pressure was set to 1 bar. The minor difference in the mean value indicates the success of the simulation, and the deviation is due to the large size of the protein. The pressure value changes widely during the process of MD simulation, as indicated by the large RMSF. Taking the pressure into consideration, the running average of the density is calculated and demonstrated in Supplementary Figure S2D; the expected density of the SPC/E mode is 1,008 kg/m^3^, and the experimental value is 1,000 kg/m^3^. The obtained average over the entire period is 1,008 ± 2 kg/m^3^, which is close to the two mentioned values and validates the success of the simulation process. The density values are stable over the entire course, indicating that the system is equilibrated well for both pressure and density.

MD simulation was performed to investigate the binding process of Cmpd 1 to TPH2. Supplementary Figure S3A demonstrates the RMSD value relative to the minimized and equilibrated models (black line) as well as to the cryo-EM structure of the TPH2 tetramer (red line) after docking. The RMSD levels off to ∼0.45 nm (4.5 Å) of both time series in the plot, manifesting the stability of the TPH2 complex, although slight differences exist between the two lines when t = 0, which makes sense for the impact of EM. After that, we analyzed the radius of gyration (Rg) of the complex, which is a measurement of compactness and might provide further information about its stability. The Rg value is theoretically steady for a stably folded protein, and it changes over time once the protein unfolds. From the results of the MD simulation, which is shown in Supplementary Figure S3B, the TPH2 tetramer complex is quite stable since the Rg value is basically unchanged over the course of 100 ns at 300 K. RMSF is another useful measurement to describe the stability of biomacromolecules, which instructs how much an individual residue or atom moves over the MD simulation process; a higher value usually indicates greater flexibility. The results are shown in Supplementary Figure S3C; the greatest score comes from atoms 21818 and 10908 from residue I490 (Figure 2C), as well as from atom 21803 from residue L488 in the chain A/B interface, which implies the important roles of these interface residues in maintaining the stability of the TPH2 complex. After MD simulation, we compared the conformational changes with Cmpd 1 during the simulation. The structural alignment shown in Figure 4D indicates that the C-terminal oligomerization domain has relatively larger conformational changes. When superimposed with chain A, chains B–D have a relative rotation, similar to that observed in the TPH2 crystal structure (Figure 2B). Analysis on the chain A/C major interface shows that the number of salt bridges increase from 3 to 9 for TPH2 with Cmpd 1 bound, while as control there is only five salt bridges after simulation for the TPH2 without a ligand bound. This indicates that binding of Cmpd 1 will stabilize TPH2 and serve as the activator of TPH2. We also compared Cmpd 1 with other top hits and found that binding of Cmpd 1 to TPH2 will give the largest RMSD with 3.567 Å compared with the structure before simulation (RMSD: 2.546 for the unbound form and 3.567, 2.667, 2.893, 3.437, 3.155, 2.396, 2.805, 2.585, 2.794, and 2.270 for top 1–10 hits, respectively).

### MM/PBSA Free Energy Calculation

The free energy of binding shows the suitability of the ligand transited from the solvated mode to the protein-bound mode. Models with lower free energies are convinced to be more stable and rigid than those with higher ones. In our study, we conducted free energy estimation using the approach of MM/PBSA to characterize the stability of the TPH2 complex in a semiquantitative method (Thompson et al., 2008; Cavalheiro et al., 2017; Poli et al., 2020). The binding free energies of the TPH2 tetramer are summarized in Supplementary Table S2, which basically includes the electrostatic energy (EEL), van der Waals energy (VDWAALS), and nonpolar solvation energy (ENPOLAR). The graphical result is also shown in Figure 4E, which clearly illustrates that van der Waals energy contributes most to favor the binding and interacting of Cmpd 1 to the TPH2 tetramer. To better understand the interaction of the TPH2 complex, we performed MM/PBSA decomposition analysis, demonstrated in Figure 4F, and we can see that Y446, L341, and A342 (Figure 4C) are most critical in mediating the formation of the complex owing to their highest energy contributions, which have also been deciphered to participate in the confinement of dimeric interfaces.

## Conclusion

**FIGURE 5:**
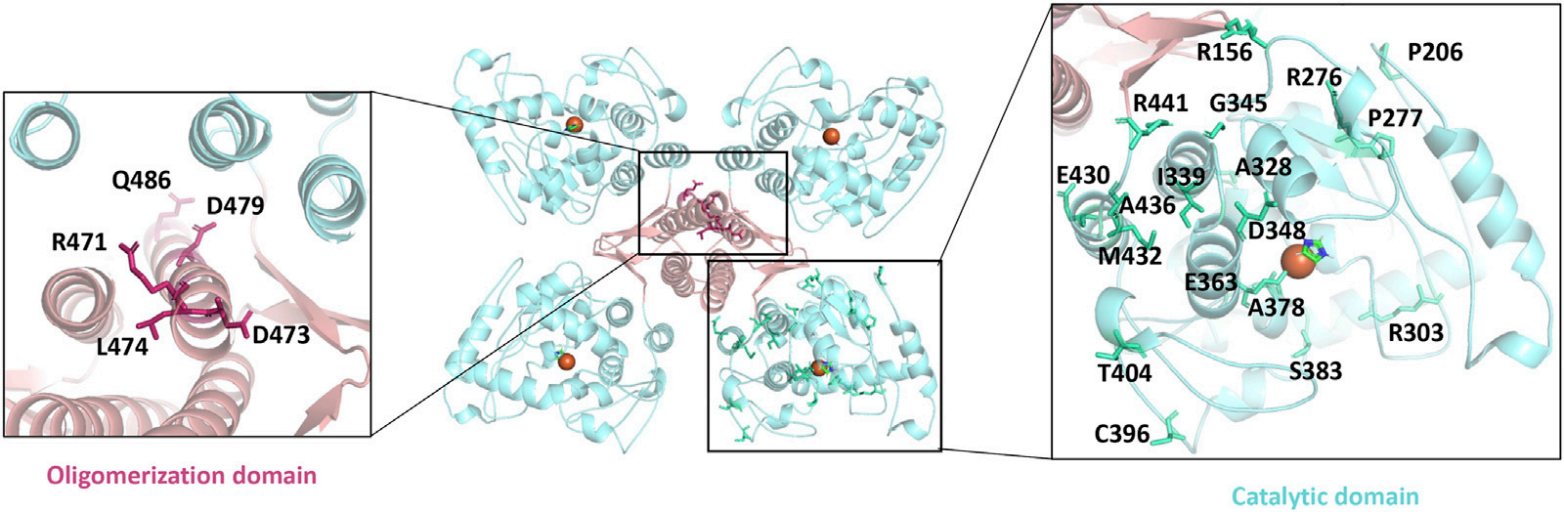
Mapping the disease-causing mutations on tryptophan hydroxylase 2 structures. The left panel shows the mutants of the oligomerization domain, and the right panel shows the catalytic domain. All mutants are shown as sticks.

Serotonin participates in various metabolic processes, and its dysregulation of expression or activation results in several types of mental illnesses including depression, autism, and bipolar disorder as well as Alzheimer’s disease, placing a huge burden on the patients and the families (Chakraborty et al., 2019; Bacqué-Cazenave et al., 2020; Takumi et al., 2020). TPH is the rate-limiting enzyme during the whole process of serotonin secretion and therefore considered as a target for the regulation of serotonin concentration (Waløen et al., 2017). In general, a decrease of serotonin in the brain and an increase in the periphery of the patients occurs simultaneously. Thus, the two main sources of serotonin are TPH2 in the brain and TPH1 in the periphery, and an activation in TPH2 and an inhibition in TPH1 are desired (Bader, 2020). Although several TPH1 inhibitors have been developed for elevating serotonin levels in peripheral tissues, the severe side effects such as impairing the activity of TPH2 hampered their clinical applications (Crane et al., 2015). In particular, there are as many as 46 disease-causing mutations of TPH2 identified to affect its folding and finally its catalytic activity (Pereira et al., 2020). The oligomerization domain and catalytic domain are two hot spots for those mutations. Some of those residues are mapped in Figure 5, including R471, D473, L474, D479, and Q486 from the oligomerization domain, as well as R156, P206, R276, P277, R303, A328, I339, G345, D348, E363, A378, S383, C396, T404, E430, M432, A436, and R441 from the catalytic domain. Besides, the redox state also impairs the function of TPH2, and the oxidation-facilitated disulfide cross-linking of C357 and C406 promotes the misfolding and aggregating that lead to the formation of high-molecular-weight aggregates with the tendency to be degraded and inhibit the enzyme activity as demonstrated *in vitro* and in the cellular level (Yohrling et al., 2000; Kuhn et al., 2011). The oxidation also obstructs the obtaining of protein *in vitro*. TPH2 activation and stabilization are hence regarded as two most meaningful and valuable directions for the treatment of psychological disorders.

In this research, we reported the cryo-EM structure of human TPH2 at 3.0 Å in the tetramer state, which explains the pathogenesis of TPH2 mutants such as P206S, R441H, and P449R and provides the fundamental information for drug design. To obtain the stabilizers and activators of TPH2, we conducted virtual screening accompanied with MD simulations, which is now a rapidly growing method for faster and cost-efficient drug discovery. The outcomes indicate that Cmpd 1 in the ZINC15 database has the greatest potential to work on it, shedding light on the development of novel drugs for the treatment of psychology disorders. Although it could represent challenges to chemically synthesize Cmpd 1, experimental investigation will be the next direction to develop novel therapies based on our study. For example, it will provide more details to investigate how Cmpd 1 could affect the dimer–tetramer equilibrium using solution measurements such as dynamic light scattering or photometry and to analyze the complex structure of TPH2 bound with Cmpd 1 by cryo-EM. The investigation on the phosphorylation and interaction of TPH2 N-terminal to 14-3-3 proteins is another route worth trying, although we still face a wide range of challenges to nail it (Winge et al., 2008; Broadbelt et al., 2012; Skjevik et al., 2014). More efforts on understanding the mechanism of the TPH2 regulatory process must be taken to foster the drug design and discovery.

## Data Availability

The original contributions presented in the study are included in the article/Supplementary Materials, and further inquiries can be directed to the corresponding authors.

## Supplementary Material

Supplementary Figure S1 Data processing of human tryptophan hydroxylase 2. Procedure of data processing was illustrated. The collected micrographs were display after motion correction. The resolution of the final map was estimated based on the golden standard FSC curves at 0.143 criteria.

Supplementary Figure S2 Energy minimization and equilibration. **(A)** variation of potential energy with energy minimization step. **(B)** variation of temperature within 100 ps. **(C)** variation of pressure within 100 ps. **(D)** variation of density within 100 ps.

Supplementary Figure S3 Molecular dynamics simulations of tryptophan hydroxylase 2 (TPH2) binding to the ligand. **(A)** root-mean-square deviation (RMSD) relative to the equilibrated system and the TPH2 complex. The black line represents the equilibrated system while the red one corresponds to the TPH2 complex. **(B)** variation of radius of gyration. **(C)** RMS fluctuation for all atoms.

Supplementary Table S1 Docking results of VinaLC between protein and ligands.

Supplementary Table S2 Energy difference for the receptor–ligand complex.
