# Supplementary material for: Cryo-EM Structure and Activator Screening of Human Tryptophan Hydroxylase 2
Source: Front Pharmacol. 2022 Aug 15;13:907437. doi: 10.3389/fphar.2022.907437 (PMC9420949; doi:10.3389/fphar.2022.907437)
Supplement: Supplementary file 1 [file Table1.docx]

**Table S1. Docking results of VinaLC between protein and ligands**

| **Compound** | **ZINC ID** | **Binding Affinity(kcal/mol)** |
| --- | --- | --- |
| Cmpd 1 | ZINC000068568685 | -10.8 |
| Cmpd 2 | ZINC000252695369 | -10.8 |
| Cmpd 3 | ZINC000013549435 | -10.8 |
| Cmpd 4 | ZINC000828320609 | -10.7 |
| Cmpd 5 | ZINC000828320608 | -10.7 |
| Cmpd 6 | ZINC000012324837 | -10.7 |
| Cmpd 7 | ZINC000004697448 | -10.7 |
| Cmpd 8 | ZINC000408973471 | -10.7 |
| Cmpd 9 | ZINC000059385960 | -10.6 |
| Cmpd 10 | ZINC000101662792 | -10.6 |
| Cmpd 11 | ZINC000005618987 | -10.6 |
| Cmpd 12 | ZINC000012416800 | -10.6 |
| Cmpd 13 | ZINC000003092279 | -10.6 |
| Cmpd 14 | ZINC000003057249 | -10.6 |
| Cmpd 15 | ZINC000100350868 | -10.5 |
| Cmpd 16 | ZINC000064890565 | -10.5 |
| Cmpd 17 | ZINC000018196121 | -10.5 |
| Cmpd 18 | ZINC000000087820 | -10.5 |
| Cmpd 19 | ZINC000013549289 | -10.5 |
| Cmpd 20 | ZINC000008616256 | -10.5 |
