# Supplementary figures and images for: Cryo-EM Structure and Activator Screening of Human Tryptophan Hydroxylase 2
Source: Front Pharmacol. 2022 Aug 15;13:907437. doi: 10.3389/fphar.2022.907437 (PMC9420949; doi:10.3389/fphar.2022.907437)

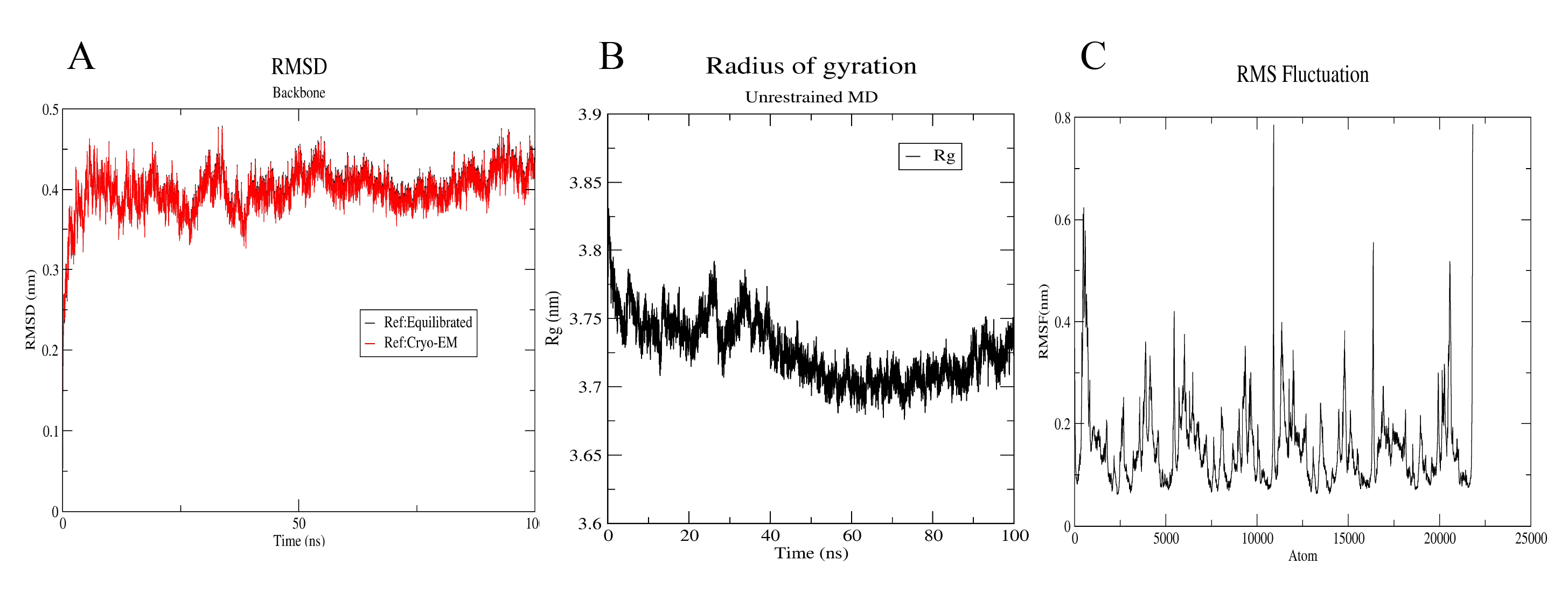

Supplement: Supplementary file 2 [file Image3.TIF]

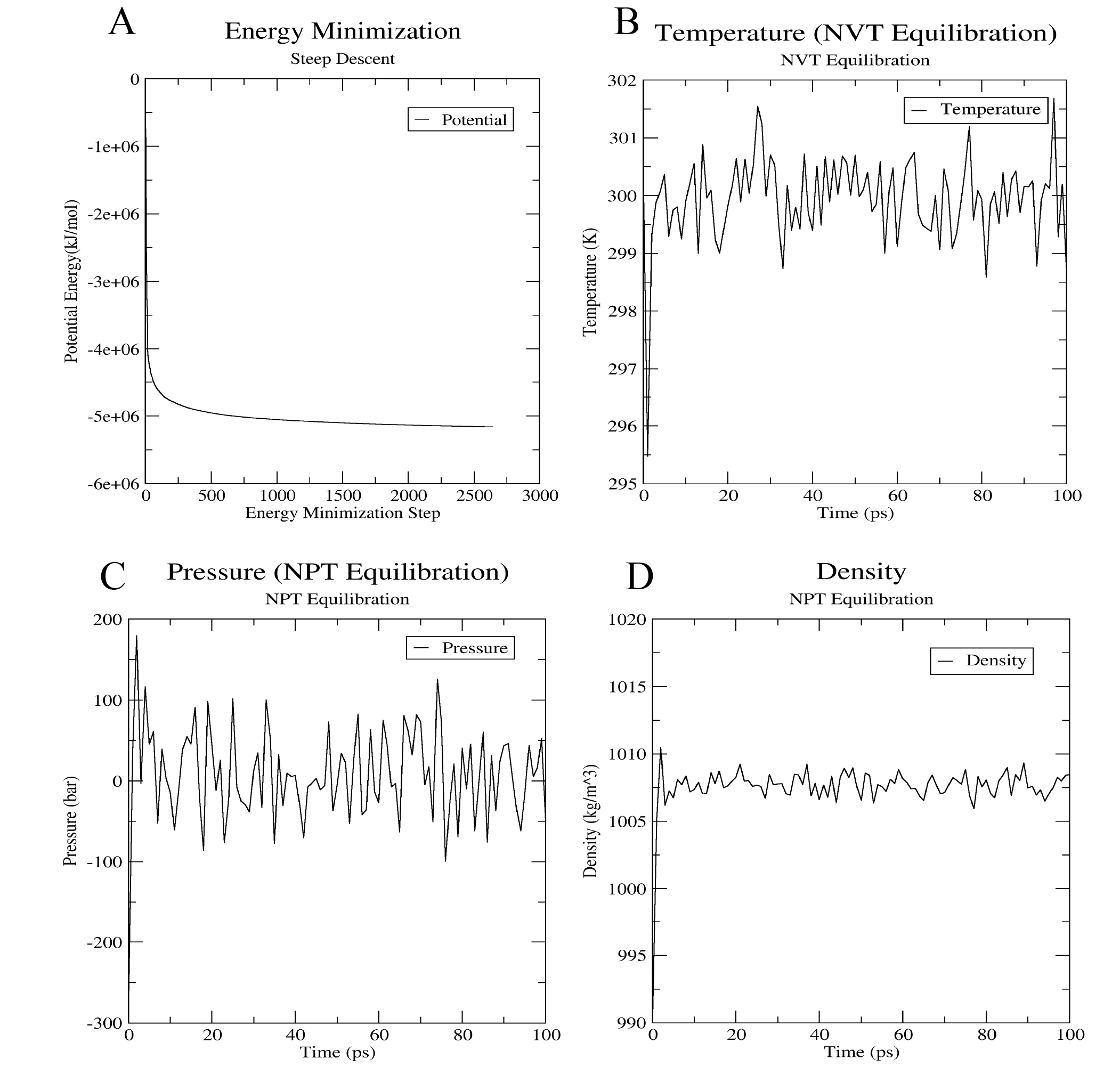

Supplement: Supplementary file 3 [file Image2.TIF]

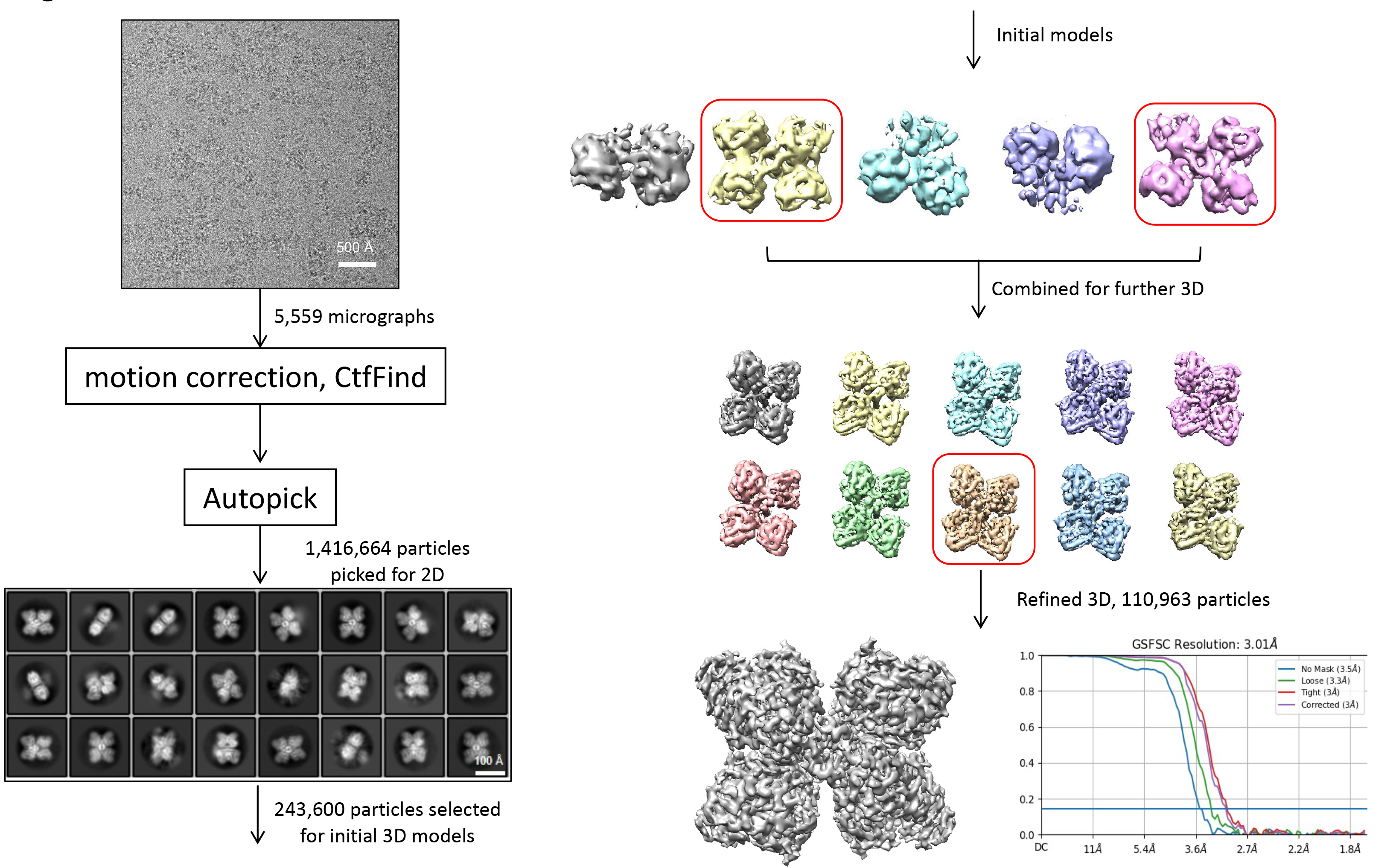

Supplement: Supplementary file 4 [file Image1.TIF]
