# Supplementary material for: Cryo-EM Structure and Activator Screening of Human Tryptophan Hydroxylase 2
Source: Front Pharmacol. 2022 Aug 15;13:907437. doi: 10.3389/fphar.2022.907437 (PMC9420949; doi:10.3389/fphar.2022.907437)
Supplement: Supplementary file 5 [file Table2.docx]

**Table S2. The energy difference for the receptor-ligand complex**

| Energy Component | Average | σ | SEM |
| --- | --- | --- | --- |
| BOND | 0.0000 | 0.0000 | 0.0000 |
| ANGLE | -0.0000 | 0.0000 | 0.0000 |
| DIHED | 0.0021 | 0.0068 | 0.0016 |
| VDWAALS | -38.5438 | 1.9189 | 0.4654 |
| EEL | -4.3477 | 4.2235 | 1.0243 |
| 1-4 VDW | -0.0000 | 0.0000 | 0.0000 |
| 1-4 EEL | -0.0000 | 0.0001 | 0.0000 |
| EPB | 48.6003 | 6.5051 | 1.5777 |
| ENPOLAR | -27.6716 | 0.6456 | 0.1566 |
| EDISPER | 54.1450 | 0.8580 | 0.2081 |
| ΔG_gas_ | -42.8895 | 5.2054 | 1.2625 |
| ΔG_solv_ | 75.0737 | 6.5775 | 1.5953 |
| ΔG_total_ | 32.1842 | 5.2256 | 1.2674 |
